# Swine liquid manure: a hotspot of mobile genetic elements and antibiotic resistance genes

**DOI:** 10.1038/s41598-020-72149-6

**Published:** 2020-09-14

**Authors:** Fengxia Yang, Bingjun Han, Yanru Gu, Keqiang Zhang

**Affiliations:** 1 Agro-Environmental Protection Institute, Ministry of Agriculture and Rural Affairs, Tianjin, 300191 China; 2 College of Resources and Environment, Northeast Agricultural University, Harbin, 150036 China

**Keywords:** Biological techniques, Environmental sciences

## Abstract

The overuse or abuse of antibiotics as veterinary medicine and growth promoters accelerates antibiotic resistance, creating a serious threat to public health in the world. Swine liquid manure as an important reservoir of antibiotic resistance genes (ARGs) has received much attention, but little information is known regarding the occurrence, persistence and fate of ARGs-associated mobile genetic elements (MGEs) in swine farms, especially their change patterns and removal in full-scale piggery wastewater treatment systems (PWWTSs). In this study, we searched the presence and distribution of MGEs and associated ARGs in swine farms, and addressed their fate and seasonal variation in full-scale PWWTSs by real-time quantitative PCR (qPCR). Our results revealed class 1 integrons, class 2 integrons and conjugative plasmids were prevalent in pig feces and piggery wastewater. A clear pattern of these MGE levels in swine liquid manure was also observed, i.e., *intI*1 > *intI*2 > *tra*A (*p *< 0.01), and their absolute abundances in winter were all higher than that in summer with 0.07–2.23 logs. Notably, MGEs and ARGs prevailed through various treatment units of PWWTSs, and considerable levels of them were present in the treated effluent discharged from swine farms (up to 10^1^–10^7^ copies/mL for MGEs and 10^3^–10^8^ copies/mL for ARGs). There were significant correlations between most ARG abundance and MGE levels (*p *< 0.05), such as *tet*Q and *tra*A (*r *= 0.775), *sul*1 and *intI*1 (*r *= 0.847), *qnr*S and *inI*2 (*r *= 0.859), suggesting the potential of ARGs—horizontal transfer. Thus the high prevalence and enrichment of MGEs and ARGs occurred in pig feces and piggery wastewater, also implicating that swine liquid manure could be a hotspot for horizontal transfer of ARGs.

## Introduction

Nowadays, antibiotics have been widely used worldwide, especially in modern animal husbandry, where they are often used as veterinary drugs for the therapy of infectious diseases, like sulfonamides, quinolones, and macrolides^1,2^. And some antibiotics are even used for growth promoters, like small sub-therapeutic doses of tetracycline, to improve the animal's immunity and increase production^3^. However, the overconsumption of these veterinary antibiotics promotes the occurrence of antibiotic resistance in food animals, and also accelerates the dissemination process of antibiotic resistance ^4,5^. Antibiotic resistance genes (ARGs), as emerging contaminants, play an important role in antibiotic resistance^6^ via various mechanisms, including enzymes that degrade or modify the antibiotic, modification of cell components like cell wall and ribosomes, and finally efflux pumps that could confer multiple resistance to bacteria^7^. Numerous studies have demonstrated that ARGs and antibiotic resistant bacteria (ARB) occurred in various farm wastes like animal feces^8–10^ and livestock wastewater^11–13^. Unfortunately, the land fertilization of livestock manure may introduce ARGs and pathogenic bacteria to farmland soil^14–16^, groundwater and surface water in the surrounding environment^17,18^. Our previous work had showed that manure fertilization increased ARGs abundance in farmland soil^19^. Thus returning animal manure to the farmland as a fertilizer represents a potential route of animal-diverted ARGs entering into environment^20–23^, although most ARGs may originate from the environment^24^. According to WHO and several recent researches, there are still many gaps in our knowledge about the environmental fate of animal-derived ARGs and its influence factors, especially regarding the change and molecular spread mechanism of ARGs during animal waste treatment processes^25,26^. Therefore, extensive information about the spread and discharge pattern of ARGs in livestock farms is needed to reduce the health risks that posed by animal-derived ARGs^25^.

It is well known that horizontal gene transfer (HGT) plays a vital role in ARGs spread^27^. Especially that the presence of antibiotics at low concentrations can accelerate horizontal transfer and dissemination of environmental ARGs^28^, which further increases the prevalence of ARGs in the environment. Animal-derived ARGs can be transferred among different organisms by HGT. And even some ARGs, present in livestock farms, could be introduced into the human pathogens via horizontal transfer^29,30^. As the components of horizontal gene pools, mobile genetic elements (MGEs) like plasmids^31,32^, and integrons^33,34^, are the cause for ARGs dissemination in environmental and human pathogens. Notably, the plasmids, especially conjugative plasmids, are one of the most important carriers of ARGs and play a significant role in the ARGs dissemination^35–38^. Conjugative plasmids belong to self-transfer plasmids, which are essential vectors for conjugation transfer of ARGs. The spread of ARGs via conjugative plasmids is common in natural environment, meanwhile, this propagation mechanism is often responsible for clinical bacterial acquired resistance as well^39^. Consequently, conjugative plasmids are crucial to facilitate ARGs horizontal transfer in environmental indigenous microbiota^35^. Besides, integrons can also promote ARGs transmission by capturing, integrating, expressing resistance gene fragments in their variable region and transferring with the aid of plasmids or transposons, which generally related closely to multidrug resistance^40–42^. And integrons carrying multidrug-resistant gene cassette have been frequently detected in the environment^43–45^. Acting as an important mechanism in the gene transfer, integrons promote the horizontal transfer of ARGs between microbes combined with mobile gene platforms^46–48^. Clearly, examining the relationships between these MGEs and ARGs can help address the problem of what role MGEs play in spreading ARGs in the environment.

In this study, two large-scale swine farms using different waste disposal systems (Farm 1 with anaerobic digestion—lagoon, Farm 2 with anaerobic digestion—aerobic biological treatment) were selected to search for MGEs and ARGs by qualitative PCR and real-time quantitative PCR. Here we concretely studied the distribution, persistence and spread pathway of MGEs and ARGs in the both swine farms, particularly their change patterns and removal in full-scale piggery wastewater treatment systems (PWWTSs), as well as to explore the potential role of MGEs in spreading ARGs in swine farming environment; We also evaluated the changes of MGEs and ARGs in summer and winter in PWWTSs. Determining this information is essential and critical to understanding and preventing the dissemination of animal-derived ARGs in the environment.

## Materials and methods

### Descriptions of swine farms

**Figure 1:**
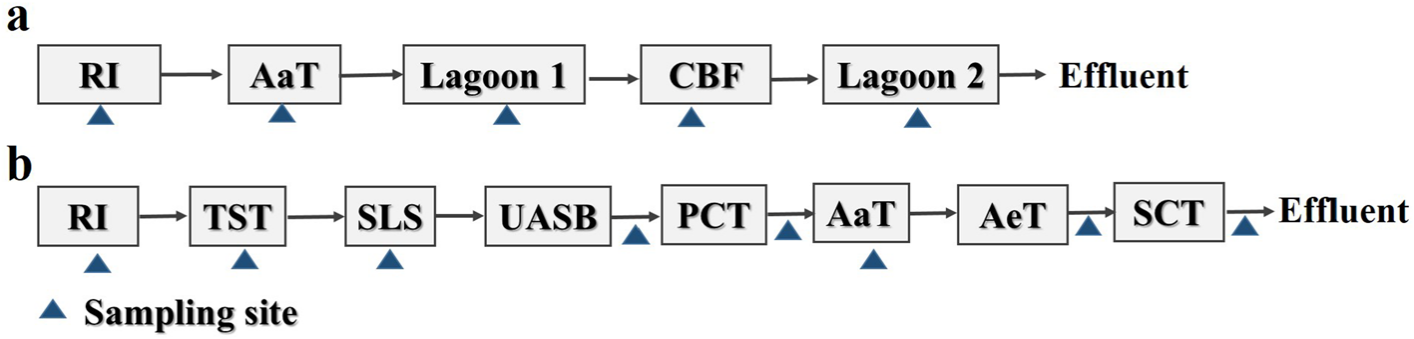
Flow chart of wastewater treatment systems in the both swine farms [(**a**) swine farm 1; (**b**) swine farm 2. *RI* raw influent, *AaT* anaerobic tank, *CBF* ceramsite biofilter, *PFR* plug flow anaerobic reactor, *TST* temporary storage tank, *SLS* solid–liquid separation, *UASB* upflow anaerobic sludge blanket, *PCT* primary clarifier tank, *AeT* aerated tank, *SCT* second clarifier tank. The blue triangles represent sampling sites].

Two large-scale swine farms (farm 1 and farm 2) in Tianjin of China, were investigated in this study. Swine farm 1 produces approximately 3,000 breeding pigs and 7,000 commercial pigs annually. There are 1,200 breeding pigs and 20,000 commercial pigs annually in farm 2. Farm 1 adopts an anaerobic digestion—lagoon treatment system, and swine farm 2 uses anaerobic—aerobic biological treatment to deepen processing piggery wastewater. The flow chart of wastewater treatment and the distribution of sampling points of the both swine farms are shown in Fig. 1. The treatment process flow chart of farm 1 contains raw influent (RI), plug flow anaerobic reactor (PFR), Lagoon 1.0, ceramsite biofilter (CBF) and Lagoon 2.0, as indicated in Fig. 1a. Farm 2 contains flow charts of raw influent (RI), primary clarifier tank (PCT), anaerobic tank (AaT), aerated tank (AeT), second clarifier tank (SCT), lagoon (effluent), as indicated in Fig. 1b. The more detail information about the processing of the both PWWTSs is listed in Table S1 of Supplemental Information.

### Swine liquid manure sampling and DNA extraction

In July (summer) and December (winter) 2017, we sampled piggery wastewater and swine feces from the two swine farms, respectively. For wastewater samples, 300 mL were collected three times continuously in the each outlet of piggery wastewater treatment stages. For pig feces (i.e., fattening pig feces, piglet feces and sow feces), they were sampled using sterile container from fresh feces on selected farms. For each fecal type, 3–5 discrete sub-samples were collected according to the each fecal pile size, and then they were freeze-dried using a freeze dryer (FD-80, Beijing Boyikang Instrument Co., Ltd., China) and mixed equally as a fecal sample. All the collected samples were placed on ice packs and then sent to laboratories for follow-up processing.

Before DNA extraction, all swine feces were dried in a freeze dryer (Tianfeng, Shanghai, China) to ensure the same low levels of water. Total DNA samples were extracted from 0.5 g of each freeze-dried sample with the FastDNA Spin Kit for Soil (MP Biomedicals, CA, USA), as indicated by the manufacturer. The samples of piggery wastewater were firstly filtered using 0.22 μm filter membranes, then the filters were put into the extraction tubes provided by FastDNA Spin Kit for Soil (MP Biomedicals, CA, USA). In the process of DNA extraction, *Escherichia coli* DH5α carrying the CESA9 gene was used as an endosomal marker for determining DNA extraction efficiency of swine feces and wastewater samples^49^. The DNA recovery rate was listed in Table S2. The extracted DNA samples were then tested for integrity using λDNA HindIII Ladder by agarose gel electrophoresis, and DNA concentration and quality of each extract were checked by BioPhotometer plus microspectrophotometry (Eppendorf, Germany). Finally, those DNA samples extracted from swine liquid manure (feces and wastewater) were stored in a 20 °C refrigerator until use.

### Qualitative PCR of MGEs and ARGs

For determining the occurrence of MGEs and ARGs, common qualitative PCR assays were conducted using the specific primers on a Biometra thermocycler (Biometra T Gradient, Germany) targeting at *intI*1 (class 1 integron-integrase) and *intI*2 (class 2 integron-integrase), *tra*A (encoding the pilin subunit of the conjugative pilus in conjugative plasmids) and eight common ARGs (*tet*O, *tet*W, *tet*Q, *sul*1, *sul*2, *oqx*B, *qnr*S and *erm*C). The detail information of primer sequences and the corresponding references are listed in Table S3. All primers of the target genes were synthesized by Sangon Biotech (Shanghai, China). The specificity of them was checked in our study with the BLAST program in Genbank to ensure the accuracy and reliability of the experiment data. PCR reaction was 25 μL mixed system, including 12.5 μL of EasyTaq PCR SuperMix (TransGen Biotech, Beijing, China), 0.5 μL of each primer (10 μM), and 0.5 μL DNA template. The PCR thermal cycle for target genes started with an initial DNA denaturation at 95 °C for 5 min, then it will be denaturated at 95 ℃ for 30 s, annealed at the temperature specified in Table S3 for 30 s, extended at 72 ℃ for 1 min, and finally extended at 72 ℃ for 7 min, with a total of 35 cycles. To examine accuracy and reproducibility of the results, all samples were subjected to PCR in duplicate, and ddH_2_O was used to instead of the DNA template for negative control for each run of PCR amplification. PCR amplified products were visualized with 1–1.5% EB (ethidium bromide) by agarose gel electrophoresis. For positive PCR products, we cloned them into pEASY-T3 vectors (TransGen Beijing, China) for sequencing verification.

### Quantitative real-time qPCR

In order to further determine the pollution level and distribution pattern of MGEs and ARGs in swine farms, the abundances of MGEs and ARGs with high detection frequencies (100%) were determined by real-time quantitative PCR (qPCR) in a 7,500 Real-Time qPCR System (Applied Biosystems). And the 20 µL qPCR reaction system contained SYBR Premix Ex Taq II (10 µL, Tli RNaseH Plus, Takara), 10 µM primer (0.4 µL for each primer, ShengGong, China), 0.4 µL ROX reference DyeII, RNase-free water (6.8 µL), and DNA samples or standard plasmid (2 µL). The amplification condition of qPCR was as follows: initial enzyme activation at 95 °C for 30 s, then 40 cycles of at 95 °C for 5 s and at 60 °C for 34 s. The standard curve for every target gene as positive control was generated as described previously^50^. Moreover, each qPCR run was repeated in triplicate, and each reaction contained a standard curve and negative control. And the specificity of qPCR products was finally checked by the melt curve in the range of 60–95 °C with an increase of 1 °C/read. The PCR assays were performed with ARGs (R^2^ 0.992–0.999) and MGEs (R^2^ 0.995–0.998) over the entire copy range with efficiencies of 90–110%. In qPCR assays of target genes, high R^2^ values and high amplification efficiencies were obtained over six orders of magnitude, which indicated the validity of these quantifications.

### Statistical analysis

The abundance of ARGs was calculated by using standard curves in Microsoft Excel 2013. The averages and standard errors for all data had been determined. The changes in the absolute abundances (the copy numbers of target genes per 1 g (dry weight) of feces or 1 mL of wastewater, copies/g DW or mL) of ARGs/MGEs and 16S rRNA gene were characterized using OriginPro 8.6 (OriginLab Corporation, USA). ANOVA analysis was used in the SPSS for Windows Release 21 (SPSS Inc. USA) to evaluate the significance of the differences among season, genes and treatment stages with gene abundance as the dependent variable, where difference was considered as significant difference when *p* < 0.05. Pearson correlation coefficient was also used to evaluate the relationship between ARGs abundance and MGEs concentrations in this study.

### Statements for the methods section and experimental protocols

We confirmed that all methods in this study were carried out in accordance with relevant guidelines and regulations, and all experimental protocols were approved by Agro-Environmental Protection Institute, Ministry of Agriculture and Rural Affairs.

## Results and discussion

### Prevalence of integrons and conjugative plasmids in swine liquid manure

Mobile genetic elements (MGEs), like conjugative plasmids, class 1 integrons, and class 2 integrons, play an important role in the migration and spread of antibiotic resistance among environmental bacteria via horizontal gene transfer pathway. In the present study, we found that *tra*A (representing conjugative plasmids), *intI1* (class 1 integrase gene) and *intI2* (class 2 integrase gene) had a detection frequency of 100% (42/42) in all pig feces and piggery wastewater samples. This result revealed that these important ARGs-associated MGEs were widely present in swine liquid manure, further implicating the high prevalence of mobile genetic elements in confined swine farms.

**Figure 2:**
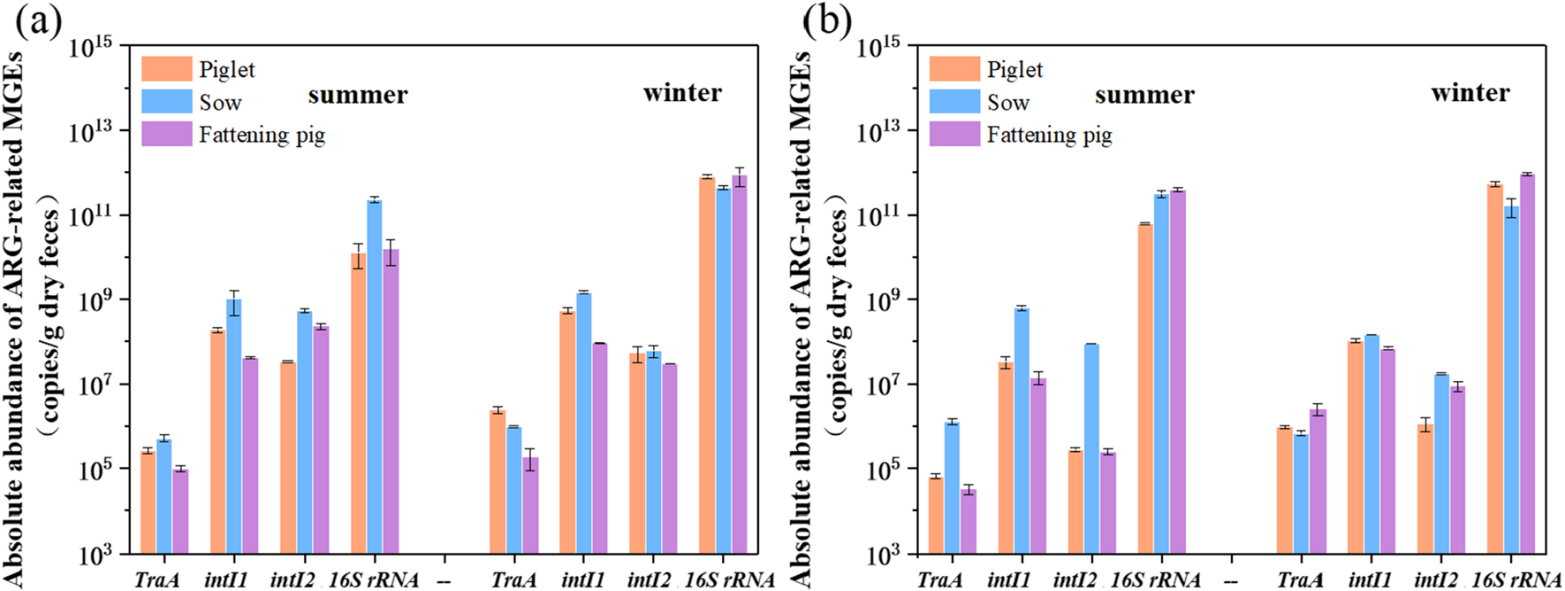
The copy numbers of ARG-related MGEs genes in pig feces samples of swine farm 1 (**a**) and swine farm 2 (**b**).

**Figure 3:**
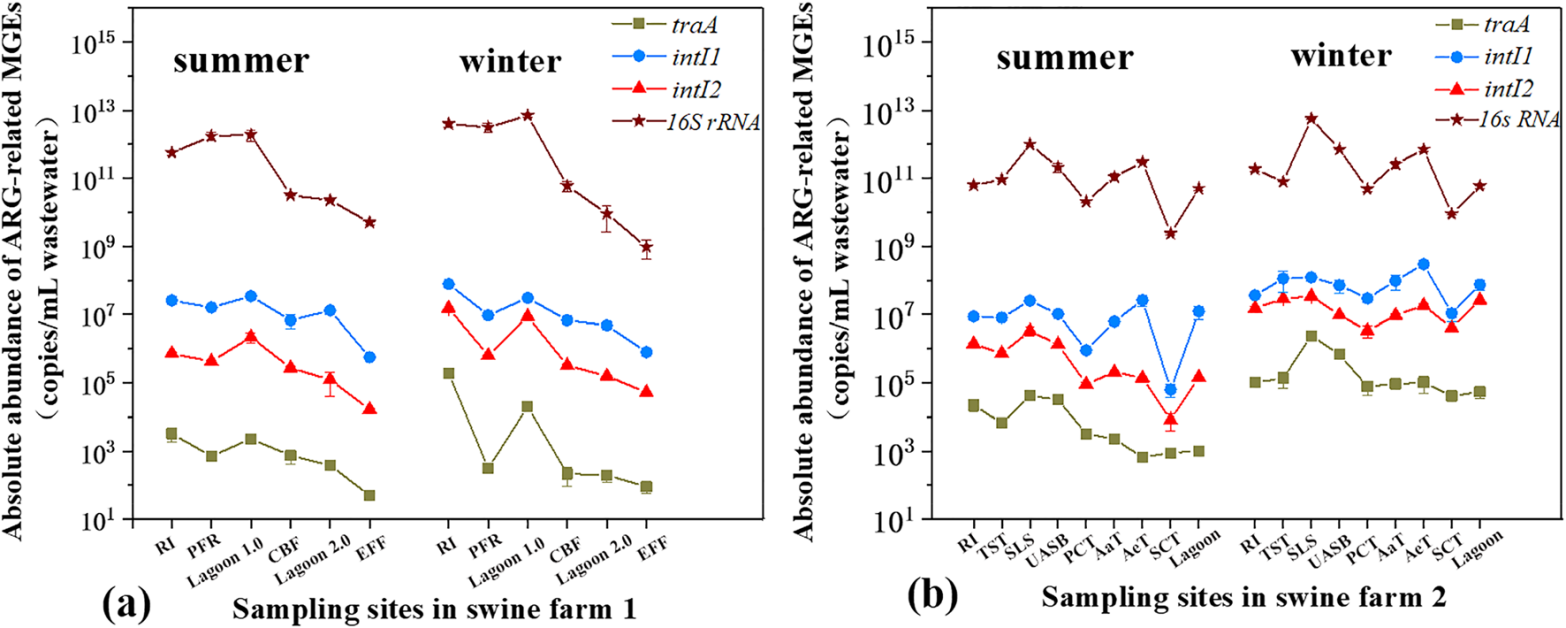
Changes in the MGEs abundance during the whole process of PWWTS 1 (**a**) and PWWTS 2 (**b**). Please refer to Fig. 1 for treatment system configuration and stage abbreviations.

For further clarifying the level and distribution of these MGEs in swine farms, the genetic marker genes of MGEs and 16S rRNA gene (a measure of total bacteria) were quantitatively analyzed by qPCR amplification. The quantitative results in this work showed that integrons were more abundant than conjugative plasmids in both swine feces, as shown in Fig. 2. In detail, the *intI1* gene levels in farm 1 and farm 2 were higher than that of *tra*A and *intI2* in the two farms, with a mean abundance of 5.57 × 10^8^ copies/g dry weight (DW) and 1.66 × 10^8^ copies/g DW, respectively, in fresh feces; while the t*raA* gene abundance was three orders of magnitude lower, at a mean level of 7.39 × 10^5^ and 9.17 × 10^5^ copies/g DW, respectively. A similar trend of these mobile genetic elements in piggery wastewater (*intI1* > *intI2* > *traA*) was also observed in this study (Fig. 3). 16S rRNA gene copy numbers as a measure of total bacteria are widely used in studies of the environment samples^51,52^. Thus we also used 16S rRNA gene copy numbers to represent the bacterial load for further normalization. The results showed that the distributions of these MGEs in swine feces and piggery wastewater were similar when relative abundance is examined (Fig. S1). The relative level of the *intI*1 gene was the highest in various swine liquid manure samples (7.45 × 10^–5^–1.49 × 10^–2^ for swine feces; 2.98 × 10^–6^–1.42 × 10^–3^ for piggery wastewater), while the investigated *tra*A gene with the lowest level occurred in swine liquid manure (8.14 × 10^–8^–2.08 × 10^–5^ for swine feces; 9.75 × 10^–10^–4.54 × 10^–6^ for piggery wastewater).

Interestingly, the MGEs abundance in sow feces were greater than that in piglet feces and fattening pig feces in both swine farms, especially in summer (Fig. 2), which may be one of the most important reasons for the higher concentrations of ARGs occurred in sow feces^49^. The mobility of ARGs in the environment mainly depends on plasmids and integrons and other MGEs^31–34,53^, and the prevalence of these shuttle vectors could accelerate the spread of ARGs in the environment. Thus the high prevalence and persistence of MGEs in swine liquid manure implied a fact that swine liquid manure could be a hotspot for horizontal transfer of ARGs.

### Diversity and abundance of associated ARGs in swine liquid manure

**Figure 4:**
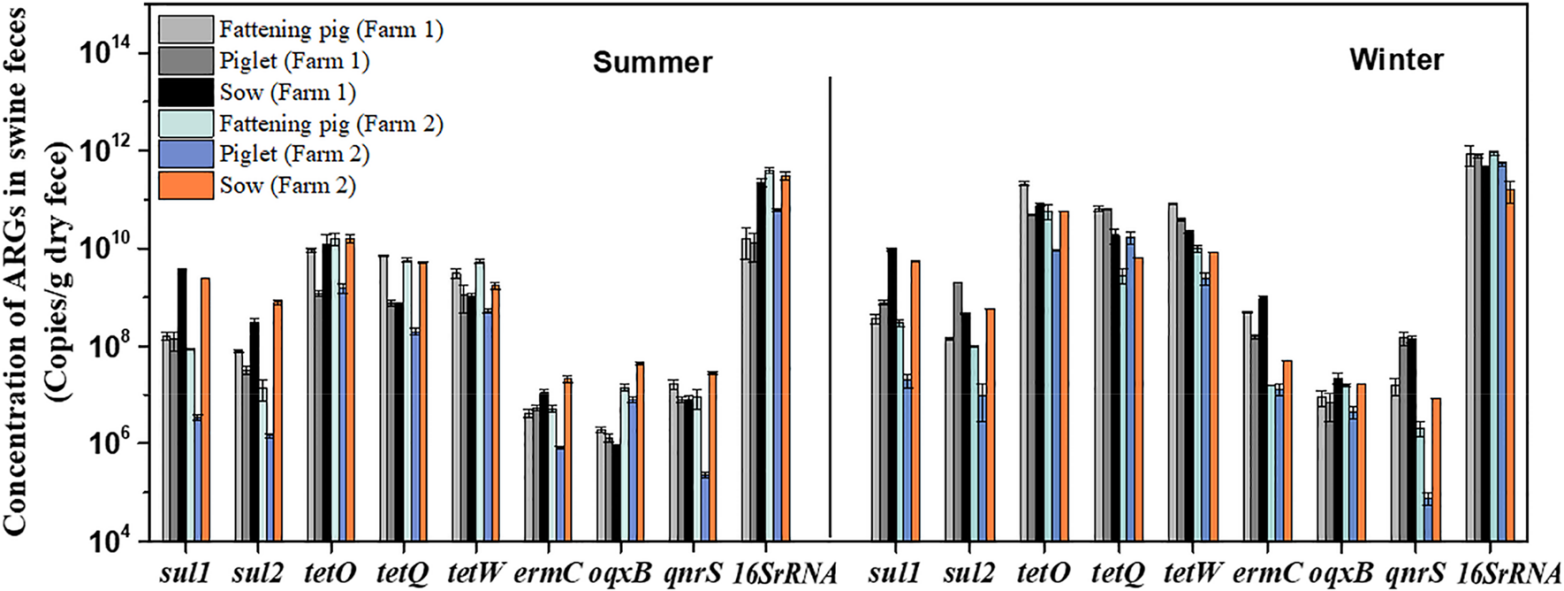
The pollution levels of familiar ARGs in pig feces of swine farm 1 and swine farm 2.

**Figure 5:**
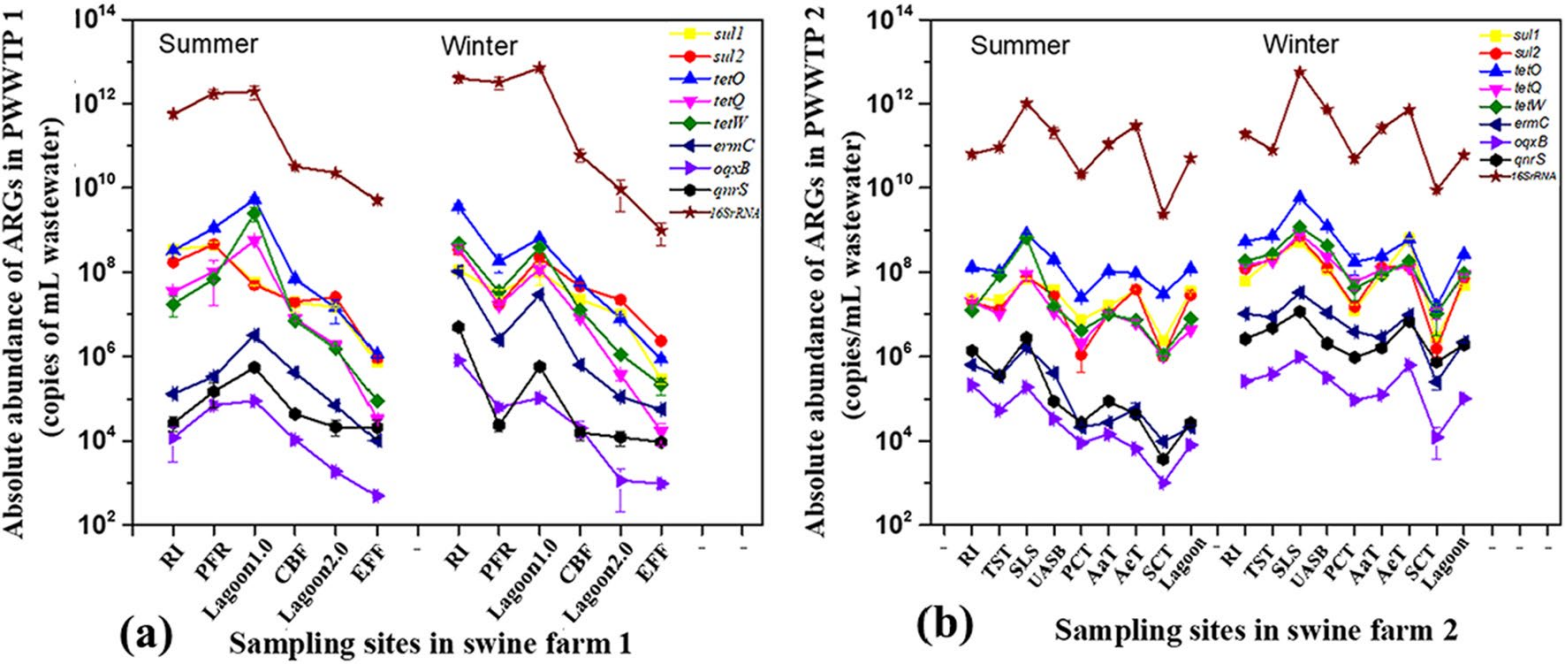
Variation in ARGs abundance during the whole process of PWWTS 1 (**a**) and PWWTS 2 (**b**). Please refer to Fig. 1 for treatment system configuration and stage abbreviations.

To characterize the MGEs-associated ARGs in detail, we quantified eight common ARGs (*sul*1, *sul*2, *tet*O, *tet*W, *tet*Q, *erm*C, *qnr*S and *oqx*B) in swine liquid manure by qPCR with specific primers that are based on known resistance genes. Although the qPCR method is not suitable for discovering novel ARGs^54^, it has been confirmed that that method is an efficient tool for studying the occurrence and abundance of ARGs, especially suitable for the exploration of the fate and migration patterns of ARGs in the environment^55–57^. Thus we mainly used qPCR to track ARGs in swine farms. The quantitative results of the present revealed that eight common ARGs were detected in all pig feces and prevailed through the full-scale PWWTSs in both swine farms (as shown in Figs. 4 and 5), demonstrating the high prevalence of ARGs and their host microbes in the two swine farms. Moreover, numerous studies also implied that swine liquid manure is a huge reservoir of ARGs^12,49,58–60^. Among these investigated ARGs, the levels of *tet*-ARGs (*tet*O, *tet*W and *tet*Q) were found to be the highest, ranging from (2.04 ± 0.35) × 10^8^ to (2.14 ± 0.21) × 10^11^ copies/g dry weight (DW) in pig feces in both swine farms, followed by *sul*-ARGs (*sul*1 and *sul*2) with a concentration range from (1.14 ± 0.13) × 10^6^ to (9.61 ± 0.59) × 10^11^ copies/g DW. While the concentration of the macrolide resistance gene (*erm*C) was comparable to those of quinolone resistance genes (qnrS and *oqx*B) (10^4^–10^8^ copies/g DW), which were lower than *sul*-ARGs and *tet*-ARGs. Of particular concern is that a similar trend of ARGs in piggery wastewater was also found in this study (Fig. 5), that is, the absolute abundance of these genes followed the order of *tet*-ARGs > *sul*-ARGs > *erm*-ARGs > *qnr*-ARGs in wastewater. Among these target ARG subtypes, *tet*O had the highest concentration in both seasons with a mean level of 1.94 × 10^9^ copies/mL in raw influent of swine farm 1 and 3.32 × 10^8^ copies/mL in swine farm 2, while *oqx*B had the lowest concentrations at 2.38 × 10^5^ copies/mL and 4.17 × 10^5^ copies/mL, respectively. Remarkably, in both swine farms, the abundances of *sul*-ARGs in sow feces were higher than that in piglets and fattening pigs (Fig. 4), probably due to the differences in dietary factors and sulfonamide antibiotic usage in different pig’s categories, such as dosage and frequency. Plasmid as an important shuttle vector of ARGs usually has two types, i.e., high-copy number plasmids and low-copy number plasmids in bacteria^61^. Although ARGs may be present e.g. on some high-copy plasmids^62,63^, it is not affect our absolute quantitative results and conclusion. Nevertheless, the fact that if ARGs are present on high-copy plasmids may overestimate their relative abundance in swine waste, which is a possible limitation of this work. Even so, the qPCR data of ARGs in swine farms were also normalized by the 16S rRNA gene copy numbers in our work, whereas we found that the distribution pattern and trend of ARGs were similar to those of absolute abundance data in swine liquid manure (Fig. S2). The *tet*O relative level was also the highest in various feces (6.42 × 10^–2^–5.79 × 10^–1^), while the investigated quinolone-resistance genes with the lowest level (1.46 × 10^–7^–1.08 × 10^–3^) occurred in pig feces.

Comparing the removal of ARGs in both swine farms, the removal effect of anaerobic digestion-lagoon system on ARGs is better than that of anaerobic–aerobic (A/O) biological treatment. Particularly, the removal range of ARGs was 0.10–4.35 logs in PWWTS1, while it was only ranged from − 0.16 to 1.72 logs removal in PWWTS2. Meanwhile, we also found that ceramsite biofilter (CBF) of PWWTS1 is the key for removing ARGs of wastewater in swine farm 1. This may be related to the fact that the ceramsite layer adsorbs and traps the vast majority of suspended matter and antibiotic resistant bacteria in piggery wastewater^64–66^. However, the A/O biological treatment system adopted by swine farm 2 is unfavorable for ARGs removal in piggery wastewater. As the inoculation of activated sludge during A/O biological treatment can increase the abundance and diversity of bacteria in wastewater, which facilitate the proliferation and dissemination of ARGs in piggery wastewater^67–69^. But troublesomely, through the treatment system of swine liquid manure, considerable levels of these ARGs were still detected in the final effluent from both farms (10^2^–10^6^ copies/mL for PWWTS1; 10^3^–10^8^ copies/mL for PWWTS2). Obviously, the persistence of ARGs in the treated wastewater posed a high risk of ARGs releasing into the receiving river and farmland environment following wastewater discharge and fertilizer irrigation.

### Variation and fate of MGEs and associated ARGs in PWWTSs

In the full-scale PWWTSs of both swine farms, changes of ARG and MGE concentrations in various treatment stages were determined to characterize the trends during piggery wastewater treatment processes. Variation in the abundances of MGE gene (*intI1*, *intI2* and *traA*) and ARGs (*sul*1, *sul*2, *tet*O, *tet*W, *tet*Q, *erm*C, *qnr*S and *oqx*B) in the full-scale PWWTSs are shown in Figs. 3 and 5. On the whole, the absolute abundance of MGEs genes and associated ARGs increased greatly through biological disposal stages (i.e., anaerobic digestion/aerobic fermentation), and follow-by decreased with physical treatment (i.e., filtration of PWWTS 1 and settlement of PWWTS 2). Evidently, the trend of selected individual MGEs genes was similar to that of the investigated ARGs (Figs. 3a and 5a; Figs. 3b and 5b). And mostly, MGEs level in the two PWWTSs was positive correlated with ARGs abundance (Table 1), indicating the potential of ARGs-horizontal transfer. Although correlations did not prove causation, these findings suggested that enhancing the elimination of plasmids and other MGEs in pre-treatment units, so as to alleviate their release to PWWTSs, may mitigate the proliferation of ARGs in swine farms.

**Table 1:** The correlation between MGEs and ARGs in swine liquid manure.

ARGs	*sul1*	*sul2*	*tetO*	*tetQ*	*tetW*	*ermC*	*oqxB*	*qnrS*
**MGEs**								
*traA*	0.506**	0.726**	0.675**	0.775**	0.374*	0.360*	0.717**	0.792**
*intI1*	0.847**	0.410*	0.300	0.419*	0.194	0.293	0.702**	0.743**
*intI2*	0.542**	0.635**	0.486**	0.644**	0.276	0.429*	0.782**	0.859**

*Correlation is significant (*p* < 0.05).

**Correlation is highly significant (*p* < 0.01).

However, the effects of various treatment processes on the abundances of MGEs and ARGs were different in the two farms. For instance, through plug flow anaerobic reactor (PFR), the concentrations of *sul*1and *sul*2 in summer were 0.2 fold and 1.7 folds higher than that in the previous stage, respectively; while their abundances increased by 1.2 folds and 8.2 folds through conventional anaerobic tank in summer, respectively. The above results implied that these conventional biological disposal units were a large repository of MGE genes and an important place of ARGs proliferation in PWWTSs. Our previous study revealed that the traditional biological units were an important stage as well for the proliferation of high-risk *bla* gene in PWWTSs^49^. It is noteworthy that most of the investigated MGEs and ARGs also showed a considerable increase in Lagoon 1 with a bacteria-algal symbiosis system in PWWTS 1, especially in summer (for example, for *tet*-ARGs, 15 folds–144 folds greater than that in raw wastewater; and 0.3 fold–2.0 folds for most MGE genes) (Figs. 3a and 5a). This is because that the algae in Lagoon 1.0 can serve as a secondary habitat for bacteria, while also protecting them from adverse environmental factors and being beneficial to their growth^70^, and the biomass of algae and bacteria can grow together by the exchange of inorganic and organic nutrients via respiration and photosynthesis^71^. In addition, the levels of these MGEs/ARGs in the wastewater through solid–liquid separation (SLS) were also increased, mainly due to the short-term floating of microorganisms in swine liquid manure.

Fortunately, those physical treatment stages of both PWWTSs, such as CBF (ceramsite biofilter) of swine farm 1 , PCT (primary clarifier tank) and SCT (second clarifier tank) of swine farm 2 (Figs. 3b and 5b), could effectively reduce the MGEs/ARGs levels in piggery wastewater. Specifically, the MGEs levels were reduced with 0.1–1.2 logs reductions (relative to raw influent) in the PCT of PWWTS2, where the associated ARGs also had a relatively high reduction with 0.4–1.7 logs reductions; In the SCT, the MGEs gene concentrations and associated ARGs levels in wastewater were also removed (approximately 0.5–2.6 logs, relative to RI) with bacterial sedimentation in this stage (Figs. 3b and 5b). Clearly, PCT and SCT in PWWTS 2 have a good effect in removing MGE and related ARG because of reduction of bacteria in the wastewater. Previous study has shown that the settling process played a vital role in ARGs reduction^72^. Earlier studies have also revealed that sedimentation plays an essential role in bacterial removal^73,74^. More remarkably, there was also a great reduction occurred in the ceramsite filter, where ARGs and MGEs were removed by 0.4–2.5 logs and 0.5–2.0 logs in PWWTS1, respectively, mainly due to the adsorption retention of drug-resistant bacteria by ceramsite layer^64–66^. Although both PWWTSs had a certain reduction in MGE and ARG abundance in the wastewater, there is still a significant concentration of them in the treated wastewater (final effluent) (up to 10^1^–10^7^ copies/mL for MGEs; 10^2^–10^8^ copies/mL for ARGs), increasing their risk of secondary dissemination into farmland environment. Obviously, the discharge of the treated piggery wastewater following farm irrigation is a non-ignored pass-way for MGEs and ARGs entering into the agricultural soil systems. Additionally, the occurrence and persistence of MGEs and ARGs in swine liquid manure also highlights the significance and necessary of adopting prevention strategies to curb the spread of antibiotic resistance, as microbial-rich pig waste with long-term exposure to sub-inhibitory antibiotic level is very conducive to the horizontal transfer of antibiotic resistance in swine farms, which was similar to other reports on other environmental medium^45,70,75,76^.

### Seasonal changes of MGEs and associated ARGs in swine farms

In the present study, we further evaluated the impact of seasonal fluctuation on MGEs and associated ARGs discharges. The trend of MGEs in summer and winter is similar to that of ARGs, the overall levels of them in winter were greater than that in summer, with change range of 0.07–2.23 logs for MGEs and 0.01–2.22 logs for ARGs (Figs. 3 and 5). Particularly, in the A/O biological treatment system of swine farm 2, the absolute concentration of these common ARGs in summer ranged from (1.01 ± 0.09) × 10^3^ to (8.09 ± 0.25) × 10^8^ copies/mL in piggery wastewater, while in winter ranged from (1.22 ± 0.86) × 10^4^ to (5.91 ± 0. 31) × 10^9^ copies/mL; As for MGEs genes, their concentration in wastewater ranged from (6.67 ± 0.92) × 10^2^ to (2.63 ± 0.09) × 10^7^ copies/mL and (4.08 ± 1.23) × 10^4^ to (3.01 ± 0.01) × 10^8^ copies/mL, respectively. The distinct difference in the MGEs and ARGs abundances in both seasons suggested that the seasonal changes have an important effect on the abundances of MGEs and ARGs in swine farms. Previous researchers also reported that the levels of some ARGs in winter were higher than that in summer, such as *erm*F and *erm*B^77,78^. Notably, in all pig fecal samples of both swine farms, the levels of MGEs and ARGs in winter were all higher than that in summer, which be also the reason for the similarity in wastewater. The greater concentration of MGEs and most ARGs in winter may be related to the increasing use of veterinary antibiotics for the prevention and treatment of diseases in winter^79–81^. Furthermore, a greater number of bacteria (characterized by the 16S rRNA gene abundance) also appeared in winter (Figs. 4 and 5), which might be due to less washing and less ventilation in winter to keep piggery warm.

Additionally, the removal efficiency of MGEs and ARGs in winter is better than in summer though some separate wastewater treatment stages, where mainly relied on physical treatment, such as CBF (filtration) in PWWTS 1, and PCT/SCT (sedimentation) in PWWTS 2. The mean removal efficiencies of MGEs and ARGs were in the range of 67.8–87.4% and 60.4–97.7% in summer, while that in winter was 77.7–98.9% and 78.1–98.8% at CBF, respectively; In PCT/SCT, the abundance of MGEs and ARGs were reduced by 34.8–93.2% and 67.8–96.0% in summer, but in winter reduced by 59.2–96.4% and 65.8–99.6%, respectively. However, it is worrying that MGEs and ARGs levels were increased the lagoon applied in swine farm 2, especially in summer with increasing by approximately 0.1–195 times and 1.2–27 times, respectively. This may be related to the growth and proliferation of ARG-host bacteria in summer, but needing further research. It's worth mentioning that there were significant differences (*p* < 0.05) in the levels of MGEs and ARGs between summer and winter in both PWWTSs, which may be not only be limited to antibiotic usage, but also related to other processes that occurred in the treatment reactors or pipelines, like ecological processes^25^. In practice, some environmental factors connection with the season (e.g., the temperature) have been shown to affect the microbial growth and the number of resistant bacteria^82–84^. Thus the changes in some small environmental factors that could disturb the number and community of antibiotic-resistant bacteria, can also cause differences in MGEs and ARG levels.

## Conclusion

This study provided a comprehensive insight in the occurrence, abundance and fate of MGEs and ARGs occurring in Chinese large-scale swine farms with different piggery wastewater treatment systems. We found that class 1 integrons were the dominant MGEs in swine liquid manure, and conjugative plasmids were also prevalent in swine liquid manure. More worryingly, MGEs and ARGs were confirmed to prevail through the whole PWWTSs, further raising the risk of swine-derived MGEs and ARGs propagating into the farmland environment. This study is important, because it demonstrated that swine liquid manure served as a large reservoir of MGEs/ARGs, and suggested swine liquid manure may be a hotspot for horizontal transfer of ARGs. Meanwhile, our results also highlighted that these persistent MGEs in livestock farming environment need more attention, since their presence raised the dissemination risk of ARGs in the agricultural environment. Thus more studies are needed to further monitor the propagation of these MGEs and associated ARGs in livestock products and food crops and to explore their harm to human health throughout the food chain.

## Supplementary information

Supplementary Information.
