# Supplementary material for: Swine liquid manure: a hotspot of mobile genetic elements and antibiotic resistance genes
Source: Sci Rep. 2020 Sep 14;10:15037. doi: 10.1038/s41598-020-72149-6 (PMC7490410; doi:10.1038/s41598-020-72149-6)
Supplement: Supplementary file 1 — Supplementary Information. [file 41598_2020_72149_MOESM1_ESM.doc]

**Supplemental information for the**

**Swine liquid manure: a hotspot of mobile genetic elements and antibiotic resistance genes**

Fengxia Yang^a^, Bingjun Han^a^, Yanru Gu^b^, Keqiang Zhang ^a^,*

**Table S1**. Detail information of the two piggery wastewater treatment systems

| Swine farm | Treatment process | Flow  (m^3^/day) | Working temperature (℃) | Individual flow (m^3^/day) | Working volume (m^3^) | HRT (h) |
| --- | --- | --- | --- | --- | --- | --- |
| Farm 1 | PFR | 35 | N.C. | 35 | 300 | 206 |
|  | Lagoon 1 |  | N.C. | 35 | 360 | 246 |
|  | CBF |  | N.C. | 35 | 350 | 240 |
|  | Lagoon 2 |  | N.C. | 35 | 400 | 274 |
| Farm 2 | TST | 120 | N.C. | 120 | 126 | 25 |
|  | UASB |  | 35 | 120 | 365 | 73 |
|  | PCT |  | N.C. | 120 | 58 | 12 |
|  | AaT |  | N.C. | 120 | 58 | 12 |
|  | AeT |  | N.C. | 120 | 58 | 12 |
|  | SCT |  | N.C. | 120 | 58 | 12 |
|  | Lagoon |  | N.C. | 120 | 465 | 93 |

N.C. not controlled; PFR, plug flow reactor; CBF, ceramsite biofilter; TST, temporary storage tank; SLS, solid-liquid separation, UASB, upflow anaerobic sludge blanket; PCT, primary clarifier tank; AaT, anaerobic tank; AeT, aerated tank; SCT, second clarifier tank

**Table S2.** DNA extraction efficiency of DNA extracted from water samples

| Sample sites | | Stages in PWWTSs | | | | | | | | | | |
| --- | --- | --- | --- | --- | --- | --- | --- | --- | --- | --- | --- | --- |
| **Swine farm 1** | | RI | PFR | Lagoon1.0 | CBF | Lagoon2.0 | | EF | |  | |  |
| DER^a^ (%) | Summer | 58±1.2 | 54±2.3 | 52±4.2 | 56±3.1 | 71±4.6 | | 78±2.8 | |  | |  |
|  | Winter | 58±2.0 | 55±2.0 | 53±3.7 | 55±3.4 | 73±4.0 | | 79±2.2 | |  | |  |
| **Swine farm 2** | | RI | TST | SLS | UASB | PCT | AaT | | AeT | | SCT | Lagoon |
| DER (%) | Summer | 58±1.8 | 57±3.6 | 59±2.0 | 54±1.3 | 64±1.8 | 57±2.3 | | 57±3.7 | | 66±1.7 | 72±2.0 |
|  | Winter | 59±1.6 | 57±3.4 | 58±2.9 | 53±2.4 | 63±2.9 | 56±3.4 | | 59±3.2 | | 65±1.8 | 70±2.8 |

a: DNA extraction recovery (%); Abbreviations: RI, raw influent; PFR, plug flow reactor; CBF, ceramsite biofilter; TST, temporary storage tank; SLS, solid-liquid separation, UASB, upflow anaerobic sludge blanket; PCT, primary clarifier tank; AaT, anaerobic tank; AeT, aerated tank; SCT, second clarifier tank; EF, the final effluent.

**Table S3. q**PCR primers and their related parameters used in this study

| Target gene | Primer sequence  (5′-3′) | Annealing temperature (°C) | Reference |
| --- | --- | --- | --- |
| *sul*1 | | F-CGCACCGGAAACATCGCTGCC | | --- | | R-TGAAGTTCCGCCGCAAGGCTG | | 56 | 1 |
| *sul*2 | | F-TCCGGTGGAGGCCGGTATCTGG | | --- | | R-CGGGAATGCCATCTGCCTTGAG | | 55 | 1 |
| *qnr*S | F-GCAAGTTCATTGAACAGGGT  R-TCTAAACCGTCGAGTTCGGCG | 54 | 2 |
| *oqx*B | F-TCCTGATCTCCATTAACGCCCA  R-ACCGGAACCCATCTCGATGC | 60 | 3 |
| *tet*O | | F-ACGGARAGTTTATTGTATACC | | --- | | R-TGGCGTATCTATAATGTTGAC | | 45 | 4 |
| *tet*W | | F-GAGAGCCTGCTATATGCCAGC | | --- | | R-GGGCGTATCCACAATGTTAAC | | 60 | 4 |
| *tet*Q | | F-AGAATCTGCTGTTTGCCAGTG | | --- | | R-CGGAGTGTCAATGATATTGCA | | 60 | 4 |
| *emr*C | | F-GAAATCGGCTCAGGAAAAG | | --- | | R-TAGCAAACCCGTATTCCACG | | 57 | 5 |
| intI1 | | F- GGCTTCGTGATGCCTGCTT | | --- | | R- CATTCCTGGCCGTGGTTCT | | 55 | 6 |
| *intI2* | F- TTATTGCTGGGATTAGGC | 58 | 7 |
|  | R- ACGGCTACCCTCTGTTATC |  |  |
| *traA* | F-AAAGAATTCGAAATTGAGGTAACTTATGAATGC | 58 | 8 |
|  | R-CCCAAGCTTCGTTTTATTTCCTGTCAGAG |  |  |
| 16S rRNA | | F-CGGTGAATACGTTCYCGG | | --- | | R-GGWTACCTTGTTACGACTT | | 55 | 9 |

**
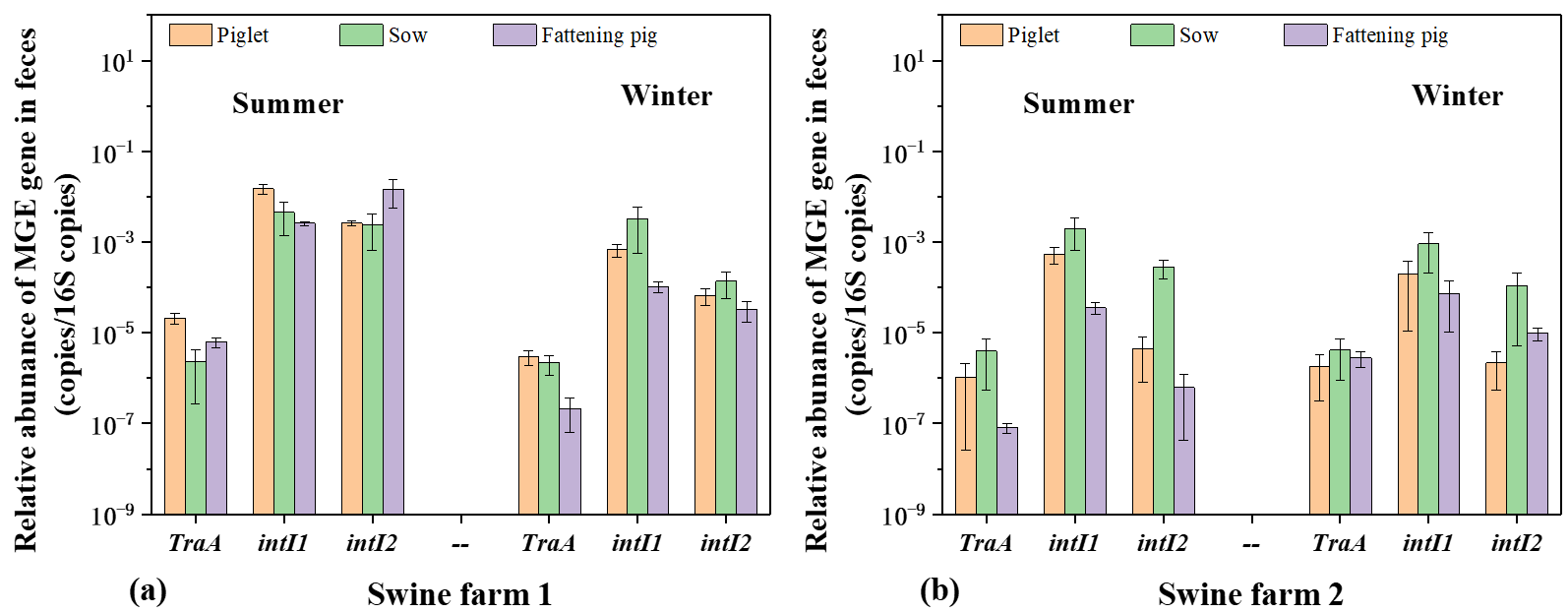
**


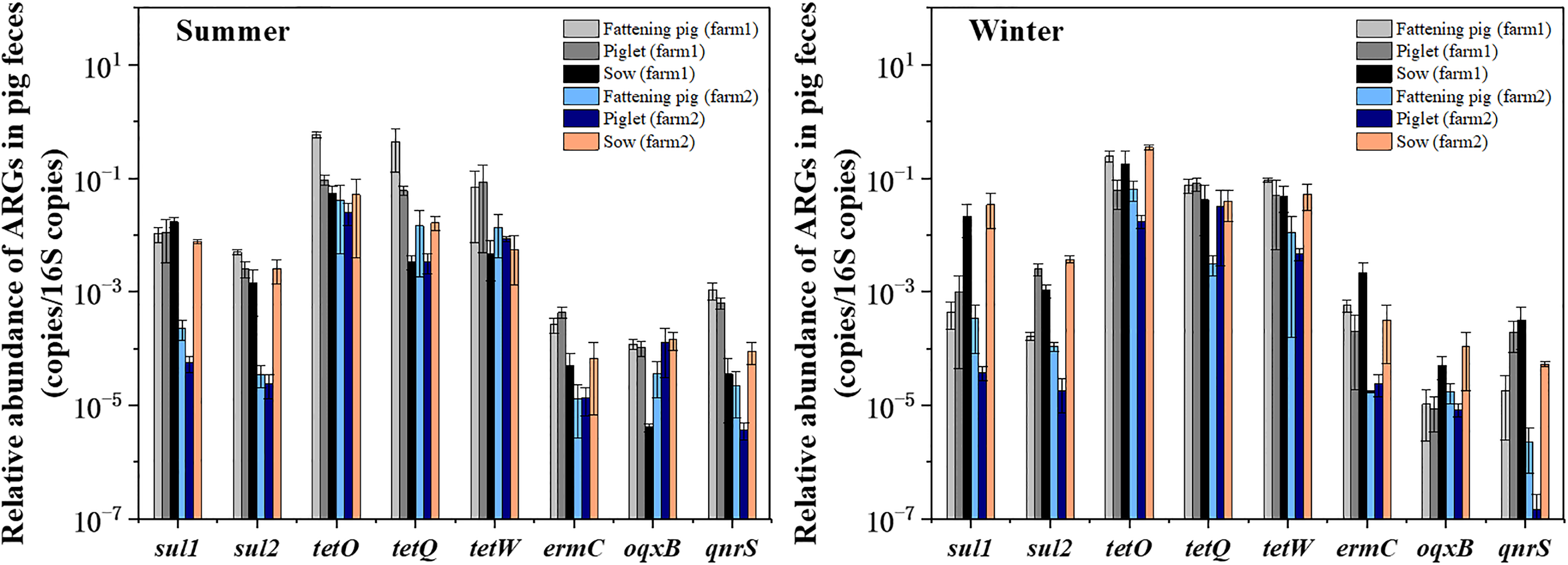
**Fig. S1** Relative abundance of MGE genes in pig fecal samples of both swine farms

**Fig. S2** Relative abundance of common ARGs in pig fecal samples of both swine farms

**References**

1. Pei, R. et al. Effect of River Landscape on the sediment concentrations of antibiotics and corresponding antibiotic resistance genes (ARG). Water. Res. 40, 2427-2435 (2006).

2. Cattoir, V. et al. Multiplex PCR for detection of plasmid-mediated quinolone resistance qnr genes in ESBL-producing enterobacterial isolates. J. Antimicrob. Chemother. 60, 394-397 (2007).

3. Kim, H.B. et al. oqxAB encoding a multidrug efflux pump in human clinical isolates of Enterobacteriaceae. Antimicrob. Agents Chemother. 53, 3582-3584 (2009).

4. Aminov, R.I. et al. Molecular ecology of tetracycline resistance: development and validation of primers for detection of tetracycline resistance genes encoding ribosomal protection proteins. Appl. Environ. Microbiol. 67(1), 22-32 (2001).

5. Mu, Q.H. et al. Occurrence of sulfonamide-, tetracycline-, plasmid-mediated quinolone-and macrolide-resistance genes in livestock feedlots in Northern China. Environ. Sci. Pollut. Res. 22, 6932-6940 (2015).

6. Luo, Y. et al. Trends in antibiotic resistance genes occurrence in the Haihe River, China. Environ Sci Technol 44, 7220-7225 (2010).

7. Goldstein, C. et al. Incidence of class 1 and 2 integrases in clinical and commensal bacteria from livestock, companion animals, and exotics. Antimicrob. Agents Chemother. 45(3), 723-726 (2001).

8. Ghigo, J.M. Natural conjugative plasmids induce bacterial biofilm development. Nature 412,442 (2001).

9. Suzuki, M.T. et al. Quantitative analysis of small-subunit rRNA genes in mixed microbial populations via 5'-nuclease assays. Appl. Environ. Microbiol. 66, 4605-4614 (2000).
